# Microbial Community Succession and Its Environmental Response in Bighead Carp (*Hypophthalmichthys nobilis*) Muscle During Low-Temperature Modified Atmosphere Transportation

**DOI:** 10.3390/foods15193430

**Published:** 2026-09-25

**Authors:** Yan Dan, Zhen Liu, Shuang Li, Chongjiang Hu, Yuankun Chen, Yan Zeng, Yibo Zhang

**Affiliations:** 1 Chongqing Fishery Sciences Research Institute, Chongqing 400020, China; danyan1154@outlook.com (Y.D.);; 2 Animal Microecology Institute, College of Veterinary, Sichuan Agricultural University, Chengdu 611130, China

**Keywords:** bighead carp, cold-chain transportation, modified atmosphere packaging, microbial diversity, microbial community succession

## Abstract

Microbial community succession contributes to quality changes in fresh freshwater fish during cold-chain transportation. In this study, bighead carp (*Hypophthalmichthys nobilis*) was used as the research material. Using 16S rRNA high-throughput sequencing, we characterized microbial community dynamics in fish muscle after 12 h and 24 h of simulated transportation under combined low-temperature (0 °C, 4 °C, 8 °C) and gradient CO_2_ modified atmosphere packaging (MAP) treatments. We further identified key environmental factors associated with shifts in bacterial communities during cold-chain logistics. The results showed that transportation temperature was the primary factor structuring microbial communities in bighead carp muscle, with stronger effects than MAP composition and transportation duration. Under 8 °C temperature conditions, low-to-moderate CO_2_ MAP corresponded with elevated microbial diversity and spoilage-associated succession, alongside enrichment of spoilage taxa including *Pseudomonas*. High-CO_2_ (80%) MAP altered community composition and reduced community complexity under low-temperature conditions, and the community changes exhibited a time-dependent pattern during extended transport. The combined treatment of 0 °C and 80% CO_2_ MAP generated distinct microbial succession profiles within 24 h and lowered the relative abundance of *Pseudomonas*. This study described the response patterns of bighead carp muscle microbiota to coupled low-temperature and MAP treatments and characterized community succession during cold-chain transport. The findings provide microbiota-based references for understanding microbial variation in freshwater fish cold-chain logistics.

## 1. Introduction

Aquaculture is one of the fastest-growing food industries worldwide. It supplies more than ten essential nutrients for human beings and represents an eco-friendly sector for safeguarding food security and dietary nutrition [1]. China ranks first in global aquaculture, with a well-established freshwater aquaculture system and substantial production capacity. As a major commercial freshwater fish in China, bighead carp (*Hypophthalmichthys nobilis*) maintains a stable annual output of 3.0–3.5 million tons, supporting a huge industry and broad consumer market [2,3,4]. Restricted by the geographical concentration of major production zones along the Yangtze and Pearl River basins, production and consumption regions for bighead carp largely overlap, creating notable bottlenecks for cross-regional and long-distance distribution of fresh products, where flesh quality is vulnerable to transport conditions [5]. Bighead carp muscle is characterized by high moisture and protein content, tender texture, and active endogenous metabolic enzymes. During cold-chain storage and transportation, fish fillets are highly susceptible to environmental disturbances such as temperature fluctuations, which induce shifts in microbial communities and accelerate flesh deterioration. Its short shelf-life and poor transport stability greatly limit the commercial circulation of bighead carp and hinder large-scale and high-quality industrial development. Therefore, exploring microbial community responses under dynamic cold-chain conditions carries important theoretical significance and practical value for expanding circulation coverage and improving the economic benefits of the freshwater-fish industry.

Modified atmosphere packaging (MAP) is a safe physical preservation technology that has been widely applied for fresh foods [6,7,8]. By replacing the internal air and reconstructing gas proportions, MAP creates a modified gaseous microenvironment that alters microbial community dynamics and oxidative changes in food matrices. Within MAP systems, CO_2_, N_2_ and O_2_ perform distinct functions. CO_2_ is the core gas component that can shape the growth environment of most spoilage microorganisms [9]. As an inert filling gas, N_2_ maintains package shape to prevent collapse and alleviates lipid oxidation [10,11,12]. Oxygen is commonly used to retain the color of fresh meat, whereas excessive O_2_ accelerates lipid oxidation and facilitates the growth of aerobic spoilage bacteria [13]. Accordingly, precise adjustment of gas ratios tailored to aquatic raw-material properties is critical for MAP applications in fresh-food distribution.

The combined application of low-temperature storage and MAP is a promising strategy for aquatic products. Low temperature reduces microbial metabolic activity and enzymatic reaction rates, while modified atmospheres alter the microenvironment for bacterial growth. Nevertheless, temperature and gas composition do not function independently; complex interactive effects exist between them [12]. Microbial community responses under MAP show obvious temperature dependence, and identical gas ratios can lead to divergent microbial profiles under different thermal conditions. Previous studies concerning bighead carp microbial succession mainly focused on static chilled storage and non-gas preservation interventions. One study characterized microbial community variation in lightly salted bighead carp fillets under a constant 4 °C refrigeration condition and illustrated the succession pattern of dominant bacteria during stable low-temperature storage [14]. A subsequent study monitored microbiota changes in ε-polylysine-treated bighead carp fillets via both culture-dependent and culture-independent approaches, also based on fixed cold storage conditions [15]. A further study explored the effects of pomegranate peel extract on microbial composition and product quality of bighead carp fillets during static chilled storage [16]. Collectively, these prior studies focused on constant-temperature storage scenarios and chemical or biological preservation methods, while microbial community dynamics under different cold-chain transport temperatures and gradient CO_2_ MAP remain poorly understood. Most existing studies have focused on quality and microbiota changes under fixed temperatures or single gas formulations. Specifically, how different temperatures couple with gradient CO_2_ concentration to regulate microbial community structure, spoilage-associated taxa succession, and community differentiation during actual cold-chain transportation remains unclear. The interactive microbial response characteristics and time-dependent microbial variation patterns under temperature–CO_2_ combined treatment has not been systematically clarified, restricting the refinement and standardization of industrial MAP parameters for freshwater fish logistics.

16S rRNA high-throughput sequencing enables comprehensive profiling of microbial community composition, taxon relative abundance and succession dynamics in fresh food, and reveals differential responses of dominant spoilage-associated microbiota under varied treatments [17,18]. It provides a powerful tool to investigate microbial community shifts under combined low-temperature and MAP conditions. Different from previous static storage and chemical preservation studies on bighead carp, the present study simulates practical variable cold-chain transportation temperature conditions and constructed multi-gradient temperature–CO_2_ MAP treatment systems with different transport durations. We systematically investigate microbial diversity, community structure, and succession of spoilage-associated bacteria in bighead carp muscle under combined temperature–MAP treatments and identify critical drivers of community differentiation. This work describes shifts in microbial communities in response to combined low-temperature and MAP treatments and offers scientific support for the process optimization of MAP for freshwater fish cold-chain logistics.

## 2. Materials and Methods

### 2.1. Samples and Pretreatment

A total of 54 healthy bighead carp (*Hypophthalmichthys nobilis*) with an average body weight of 1.5 ± 0.2 kg were obtained from Changshou Lake, Changshou District, Chongqing, China. The fish were acclimated in a laboratory recirculating water system (Qingdao Zhongkehai Water Treatment Co., Ltd., Qingdao, Sahgndong, China) for 24 h before sampling and were anesthetized with 100 mg/L tricaiine methanesulfonate (MS-222, Sigma-Aldrich, St. Louis, MO, USA). Under aseptic conditions, the fish descaled, gilled and eviscerated rapidly. Dorsal and lateral muscle tissues were collected. Tissue surfaces were rinsed gently with sterile saline to remove blood and debris, then blotted dry using sterile filter paper. Muscle samples were cut into uniform blocks of 5.0 cm × 8.0 cm × 3.0 cm, weighing 120 ± 5.0 g each. Individual muscle blocks were sealed separately in polyethylene bags (Nasco, Fort Atkinson, WI, USA), with one block per bag. All animal handling procedures complied with the requirements of the Experimental Animal Welfare and Ethics Committee of Sichuan Agricultural University (Ethics approval No. 20230634).

The reagents used in this study included a DNA extraction kit (Beijing Bomei Biotechnology Co., Ltd., Beijing, China), an AxPIeP DNA gel extraction kit (AXYGEN, Union City, CA, USA), and FastPfu DNA Polymerase (TransGen Biotech Co., Ltd., Beijing, China).

### 2.2. Experimental Design

A three-factor experimental design was applied to simulate practical commercial cold-chain transportation conditions. Three temperature levels (0 °C, 4 °C, and 8 °C) and three CO_2_ gradient modified atmosphere packaging (MAP) formulations were set in this study, including Formula A (40% CO_2_ + 10% O_2_ + 50% N_2_), Formula B (60% CO_2_ + 10% O_2_ + 30% N_2_), and Formula C (80% CO_2_ + 10% O_2_ + 10% N_2_). These gas formulations corresponded to conventional preservation modes for aquatic products under stable low temperature, routine cold-chain logistics, and temperature-fluctuation transport conditions, respectively. Two transport durations (12 h and 24 h) were designed for all treatment groups, with three biological replicates for each group. Pre-sterilized bighead carp muscle samples were placed under the above combined temperature and MAP conditions for simulated cold-chain storage and transportation. All samples were held in calibrated portable temperature-controlled transport box (Shanghai Jingchuang Electronic Co., Ltd, Shanghai, China) titted with a temperature logger for continuous temperature recording. Temperature fluctuation was maintained within ±0.5 °C. After treatment, all samples were immediately frozen at −80 °C for subsequent 16S rRNA high-throughput sequencing of microbial communities.

### 2.3. Microbial DNA Extraction and High-Throughput Sequencing Analysis

Total microbial DNA was extracted from each sample using a TIANamp Genomic DNA kit (Tiangen Biotech Co., Ltd, Beijing, China) following the manufacturer’s instructions. DNA concentration and purity were determined by spectrophotometry. The V3–V4 hypervariable region of the bacterial 16S rRNA gene was amplified by PCR with the primer set 338F/806R [19]. The 20 μL PCR mixture contained 4 μL of 5× FastPfu buffer, 2 μL of 2.5 mmol/L dNTPs, 0.8 μL of each primer, 0.4 μL FastPfu DNA polymerase, and 10 ng template DNA. The PCR thermal profile included initial denaturation at 95 °C for 3 min, followed by 27 cycles of denaturation at 95 °C for 30 s, annealing at 55 °C for 30 s, and extension at 72 °C for 30 s, with a final extension step at 72 °C for 10 min. All PCR assays were run in technical triplicate.

PCR amplicons were purified using a gel-recovery kit and quantified with NanoDrop 2000c microvolume spectrophotometer (Thermo Fisher Scientific, Wilmington, DE, USA). Libraries were constructed using the NEXTFLEX library preparation kit (Revvity Health Sciences, Inc., Austin, TX, USA) and high-throughput sequencing was performed on the Illumina NextSeq 2000 platform (Illunina Inc., San Diego, CA, USA) by Novogene Co., Ltd. (Beijing, China). Raw sequencing reads were processed on a bioinformatic cloud platform. Paired-end reads were merged using FLASH (v 1.2.11), and quality filtering was conducted using FASTP 0.23.1. Representative OTUs were clustered at 97% sequence similarity with Vsearch (v 2.16.0), and taxonomic assignment was performed against the Silva (v 138.2) database. Alpha- and beta-diversity, community composition, and differential analysis of dominant taxa were calculated from the resulting OTU table. Spearman’s rank correlation analysis was used to evaluate associations between alpha-diversity indices and environmental variables (temperature, CO_2_ concentration, transportation duration). Beta-diversity analyses including PCoA and UPGMA clustering, Kruskal–Wallis H test for genus-level differences [20], redundancy analysis (RDA), and PICRUSt2 (v 2.5.2) functional prediction were implemented in QIIME 2 (v 2022.2) [21] and R software (v 4.0.3) to explore relationships between microbial communities and environmental variables.

### 2.4. Statistical Analysis

Statistical analyses and plotting were conducted using GraphPad Prism 10.3.0. Data are expressed as mean ± standard deviation (mean ± SD). One-way analysis of variance (ANOVA) was applied to assess differences among all combined treatment groups. RDA and Spearman’s rank correlation analysis were used to explore associations between microbial community and environmental variables. Values of *p* < 0.05 were indicated by *, *p* < 0.01 by **, and no marker was assigned for *p* ≥ 0.05 in figures.

## 3. Results and Discussions

### 3.1. Combined Temperature MAP Treatments and Microbial Community Structure

Rarefaction curves and a species accumulation boxplot were generated to assess sequencing depth and sampling adequacy (Figure 1A,B). Most rarefaction curves reached a plateau as sequencing reads increased, indicating sufficient depth to recover the bacterial taxa in each sample. The species accumulation curve showed that observed species continued to rise with additional samples, but the rate of increase slowed at approximately 45 samples. This indicated that the current sample set adequately covered the bacterial diversity in this system.

UPGMA clustering and principal coordinate analysis (PCoA) based on weighted UniFrac distance were then used to examine beta diversity among treatments. The UPGMA dendrogram together with phylum-level stacked bar chart (Figure 1C) showed that samples did not cluster consistently by temperature or transport duration. Samples C412 and C824 formed separate branches away from the main cluster. PCoA (Figure 1D) showed a similar pattern. PC1 and PC2 explained 36.60% and 14.92% of total variation, respectively. Apart from C412 and C824, samples C024 and C424 were also dispersed in the ordination space. This pattern suggested CO_2_ concentration may contribute to the separation of these samples. Temperature and transport duration showed weaker associations with sample grouping in this PCoA plot.

In aquatic cold-chain systems, transport duration generally has less influence on microbial community structure than temperature and gas composition [22]. Our results agreed with this: duration alone did not drive systematic community shifts, while changes in CO_2_ concentration corresponded with clear differences in community profiles (Figure 1D). Elevated temperature has been shown to alter microbial communities in common carp, Atlantic salmon and Atlantic cod fillets [23]. Our data further indicated that the effect of CO_2_ depends on temperature. Distinct community patterns were observed only when low temperature and high CO_2_ were combined, in line with previous work [7,24].

At the phylum level (Figure 1C), Pseudomonadota, Bacteroidota, Bacillota, Actinomycetota and Deinococcota were the dominant groups. Pseudomonadota accounted for an average relative abundance of 82.13% across all samples. This phylum includes psychrotrophic genera such as *Pseudomonas*, *Acinetobacter* and *Aeromonas*, which are commonly associated with fish spoilage. *Pseudomonas* and *Shewanella*, for instance, grow rapidly in chilled fillets and produce proteases that degrade muscle proteins [25,26]. Pseudomonadota dominance has also been reported in sea bass and silver carp [27,28]. The remaining phyla maintained low relative abundances across all treatments, likely due to weaker competitiveness in fish muscle matrix under the tested conditions [29].

### 3.2. Environmental Drivers of Microbial Communities Variation

Redundancy analysis (RDA) combined with Spearman-rank correlation analysis was applied to quantify the driving effects of temperature (Temp), CO_2_ concentration (CO_2_), N_2_ concentration (N_2_), and transport-storage duration (Time) on microbial communities in bighead carp muscle (Figure 2A). RDA1 and RDA2 accounted for 43.27% and 31.23% of total community variation, respectively, yielding a cumulative 74.50%. Arrow lengths indicate that transport-storage duration was associated with clear composition differences and that temperature was the factor most strongly associated with overall community structure across the three storage temperatures. Within each temperature level, CO_2_ differentiated the MAP treatments, while N2 exerted negligible effects. The acute angle between the temperature and CO_2_ vectors suggested that these two factors covaried in their association with community composition.

These results point to a clear hierarchical order of environmental effects. Across temperatures, storage temperature was the main determinant of community structure, while transport-storage duration served as a secondary factor driving gradual microbial turnover. Temperature is known to alter microbial metabolic rates and reshape community composition [30,31], whereas CO_2_ inhibits spoilage bacterial growth by intracellular acidification and membrane damage [31,32]. At a fixed temperature, CO_2_ acted as the key factor distinguishing different MAP groups. When both temperature and gas composition remained stable, prolonged storage did not trigger abrupt community shifts, which is consistent with the results shown in Figure 1.

In the Spearman heatmap (Figure 2B), temperature was strongly and positively associated with observed species, Chao1 and ACE indices (*p* < 0.01), indicating that warmer storage conditions increased microbial richness. CO_2_ exhibited negative correlations with alpha-diversity indices, and high CO_2_ constrained the increase in microbial richness. Transport-storage duration showed no significant correlations with any alpha-diversity metrics, and N_2_ exerted minimal influences. Combined with the RDA results, these findings demonstrate that temperature dominated the structuring of microbial community structure and richness across different storage conditions, while CO_2_ served as the core variable differentiating MAP groups under identical temperature conditions.

Higher storage temperatures corresponded with greater microbial richness and higher proportions of subordinate taxa, whereas elevated CO_2_ reduced community richness and complexity. Excess CO_2_ can also impair fish flesh quality by decreasing pH, increasing drip loss, and toughening muscle texture [33,34]. Accordingly, microbial succession during simulated cold-chain transport was comprehensively modulated by temperature, CO_2_ concentration, and storage duration. By characterizing microbial community responses to coupled low-temperature and MAP treatments, this study provides a fundamental reference for optimizing MAP parameter settings for bighead carp cold-chain logistics.

### 3.3. Alpha Diversity of Muscle Microbiota Under Combined Temperature MAP Treatments

Observed species and Shannon indices were used to assess species richness and community diversity across treatments (Figure 3A,B). The two indices showed consistent variation patterns, with temperature strongly associated with shifts in alpha diversity. Under simulated temperature fluctuation at 8 °C, both observed species and Shannon indices increased, indicating higher species richness and more complex communities. In comparison, samples held at 0 °C generally maintained lower diversity values.

Changes in alpha diversity indicated that different temperature was linked to shifts in microbial community composition during simulated cold-chain transport. Minor warming may relieve low-temperature constraints on microorganisms, allowing previously less abundant taxa to multiply and increasing community complexity, which has been associated with fish quality changes [31]. Stable low temperature was associated with reduced microbial metabolic activity and relatively simple community profile.

The association between CO_2_ and alpha diversity varied with temperature. At 8 °C, treatments A (40% CO_2_) and B (60% CO_2_) did not reduce richness or diversity, whereas treatment C (80% CO_2_) showed lower diversity indices. The combination of 80% CO_2_ and 0 °C produced the lowest observed species and Shannon values among all treatment groups. Compared with temperature and CO_2_ concentration, transport-storage duration had a weak overall association with alpha diversity, though significant differences were detected in some pairwise comparisons. For example, the Shannon index differed significantly between A012 and A812, A024, B024, and B424 (*p* < 0.05).

This temperature-dependent pattern helps explain the performance of commonly used commercial MAP. Moderate CO_2_ formulations (40–60%) widely used in practice are only associated with limited shifts in microbial communities under stable low temperatures [7,8,35]. Their ability to constrain community changes diminishes under different temperatures, and microbial community shifts are observed. In contrast, the 80% CO_2_ formulation showed some capacity to resist temperature disturbance, and combined low-temperature and high-CO_2_ conditions corresponded to reduced microbial diversity.

OTU counts across gradient CO_2_ treatments at 0 °C were further examined (Figure 3C). At 12 h of simulated transport-storage, OTU numbers did not decrease as CO_2_ concentration increased. The C012 sample (80% CO_2_, 12 h) had 501 OTUs. By 24 h, OTU counts in C024 decreased to 155, which was significantly lower than groups A and B at the same time point. These results suggested that the association of 80% CO_2_ with microbial community shifts exhibited a time-lag effect. Longer gas exposure was required to reduce species richness. Even at 0 °C, 40–60% CO_2_ did not limit the expansion of bacterial taxa over longer incubation periods.

This time-lag pattern was a key observation from this study. The effect of high CO_2_ on community richness was not immediate. Sustained exposure of 24 h was needed to produce detectable reductions in species richness. Changes in community diversity were not apparent under short-term cold-chain conditions. Meanwhile, moderate-CO_2_ formulations did not show consistent capacity to restrict community shifts even with extended treatment time [36,37]. These observations highlight that combined low-temperature and high-CO_2_ conditions are associated with altered microbial communities in bighead carp muscle under long-duration simulated cold-chain transport.

### 3.4. Genus-Level Differential Taxa and Shits in Spoilage-Associated Bacteria Communities

Kruskal–Wallis H-test was applied to identify genera with significant differences among treatment groups (Figure 4, adjusted *p* < 0.05). We focused on the dynamics of *Pseudomonas*, a well-documented spoilage-associated genus in aquatic products. At 0 °C after 12 h of simulated transport-storage (Figure 4A), the relative abundance of *Pseudomonas* in group C012 (80% CO_2_) exceeded that in A012 and B012. At this 12 h time point, high-concentration CO_2_ was not associated with reduced relative abundance of *Pseudomonas*. After 24 h of transport-storage (Figure 4B), the relative abundance of *Pseudomonas* in C024 declined and was lower than the values in A024 and B024 sampled at the same time. This pattern confirmed a time-lag effect in the association between 80% CO_2_ and shifts in *Pseudomonas* relative abundance, with measurable changes only observed after 24 h exposure. In comparison, 40–60% CO_2_ at 0 °C did not correspond to reduced relative abundance of this genus over the same duration.

*Pseudomonas* contains multiple opportunistic pathogenic strains and is recognized as a common psychrotrophic, spoilage-associated taxon in cold-chain systems for freshwater fish [38,39,40,41]. Members of this genus produce extracellular proteases and lipases that degrade protein and lipids in fish muscle. Changes in its relative abundance have been linked to quality loss in fresh bighead carp. The observed time-lag pattern for high CO_2_ had practical implications for MAP design. Changes in the relative abundance of *Pseudomonas* were not detected after short exposure periods, which may partly explain the inconsistent performance reported for commercial MAP applications.

Under the simulated temperature-fluctuation condition at 8 °C, the relative abundance of *Pseudomonas* across all groups after 12 h of transport-storage was higher than that of equivalent samples held at 0 °C (Figure 4C). Even the 80% CO_2_ treatment did not offset the rise in *Pseudomonas* relative abundance associated with increased temperature. Marked shifts in community composition were observed by the 24 h time point (Figure 4D). *Pseudomonas* was no longer the primary differentially abundant genus. *Chryseobacterium*, *Glutamicibacter* and *Brevundimonas* accounted for most intergroup differences. These results showed that different temperature was associated with increased alpha diversity and turnover of dominant spoilage-associated taxa, leading to convergence of communities with spoilage-related profiles.

These observations described microbial community changes linked to different temperatures during cold-chain transport. Temperature shifts corresponded to altered community structure, promoting increases in core spoilage-associated taxa and replacement of dominant bacterial groups [27,31]. Communities from different MAP treatments tended to develop similar spoilage-associated profiles. This suggested that CO_2_ modification alone could not counteract community shifts driven by different temperature. Combined stable low-temperature and high-CO_2_ conditions were associated with distinct patterns of spoilage-related community succession in bighead carp muscle under simulated cold-chain transport.

### 3.5. Prediction of Putative Microbial Community Functions

Putative functional profiles of microbial communities were inferred using PICRUSt2 based on 16S rRNA gene amplicon sequencing data (Figure 5). The predicted profiles indicated that temperature was associated with greater changes in microbial functional categories than modified atmosphere packaging. Predicted abundances of categories linked to bacterial growth, proteolysis and lipid degradation were higher under the 8 °C condition. The combined treatment of 0 °C and 80% CO_2_ reduced the predicted abundances of these spoilage-associated functional categories.

These predicted functional patterns were broadly consistent with observations of community structure, alpha diversity and shifts in spoilage-associated taxa. The functional data provided a predicated functional perspective on community changes under different temperature and MAP conditions. Higher temperature conditions were associated with elevated predicted metabolic potential for protein and lipid breakdown [8,30], which is commonly linked to changes in fish muscle texture and sensory properties. Combined low-temperature and high-CO_2_ conditions suppressed the predicted abundance of these spoilage-related metabolic pathways.

It should be noted that these functional profiles are only computational inferences derived from microbial taxonomic composition, rather than direct experimental measurements. 16S rRNA amplicon sequencing only yields relative taxonomic abundances. This approach cannot reflect in situ microbial metabolic activity or distinguish signals from viable and non-viable bacterial cells. Accordingly, all functional results obtained in this study represent putative microbial functional potential rather than actual metabolic performance. Future studies can integrate metagenomics, metabolomics and physicochemical indicator analyses. Multi-omics datasets will enable more robust verification of how combined temperature and MAP treatments reshape microbial metabolism and trigger quality variations in bighead carp muscle, thereby further elucidating the underlying microbial succession patterns.

## 4. Conclusions

This study characterized microbial community succession in bighead carp muscle under simulated cold-chain transportation with combined low-temperature and gradient CO_2_ MAP treatments. The 0 °C and 80% CO_2_ condition generated distinct microbial community profiles, lower complexity, and reduced relative abundance of the spoilage-associated genus *Pseudomonas* after 24 h of simulated transport. Microbial responses to high CO_2_ were both time- and temperature-dependent, and significant community alterations were only observed under stable low-temperature conditions. Elevated temperature (8 °C) reshaped microbial succession trajectories, increased microbial diversity, and promoted the turnover of dominant spoilage-associated taxa.

Moderate- and low-CO_2_ MAP (40% and 60% CO_2_) induced negligible microbial community changes and exhibited preservation effects under different temperature conditions. Temperature was the dominant environmental factor driving microbial community variation during simulated cold-chain transport, whereas CO_2_-mediated microbial regulation effects were most pronounced under stable low-temperature conditions.

Previous studies on bighead carp microbiota have mainly examined static refrigeration systems with chemical or biological preservation interventions. Differing from these static treatment studies, the present study investigated microbial succession under different transport temperatures and gradient CO_2_ MAP regulation, and identified typical time- and temperature-dependent microbial response patterns. These findings supplement fundamental information regarding microbial community dynamics during freshwater fish cold-chain logistics. Notably, this study characterized variations in bacterial community structure, taxon relative abundance, and predicted functional profiles via sequence-based analysis. These bioinformatic results are not equivalent to experimentally verified bacterial inhibition efficiency, in situ metabolic activity, or practical preservation performance. Since the conclusions were derived solely from 16S rRNA sequencing data, further integration of physicochemical and sensory quality indicators is required to comprehensively evaluate the practical application value of temperature–CO_2_ combined MAP treatments in bighead carp cold-chain preservation.

## Figures and Tables

**Figure 1:**
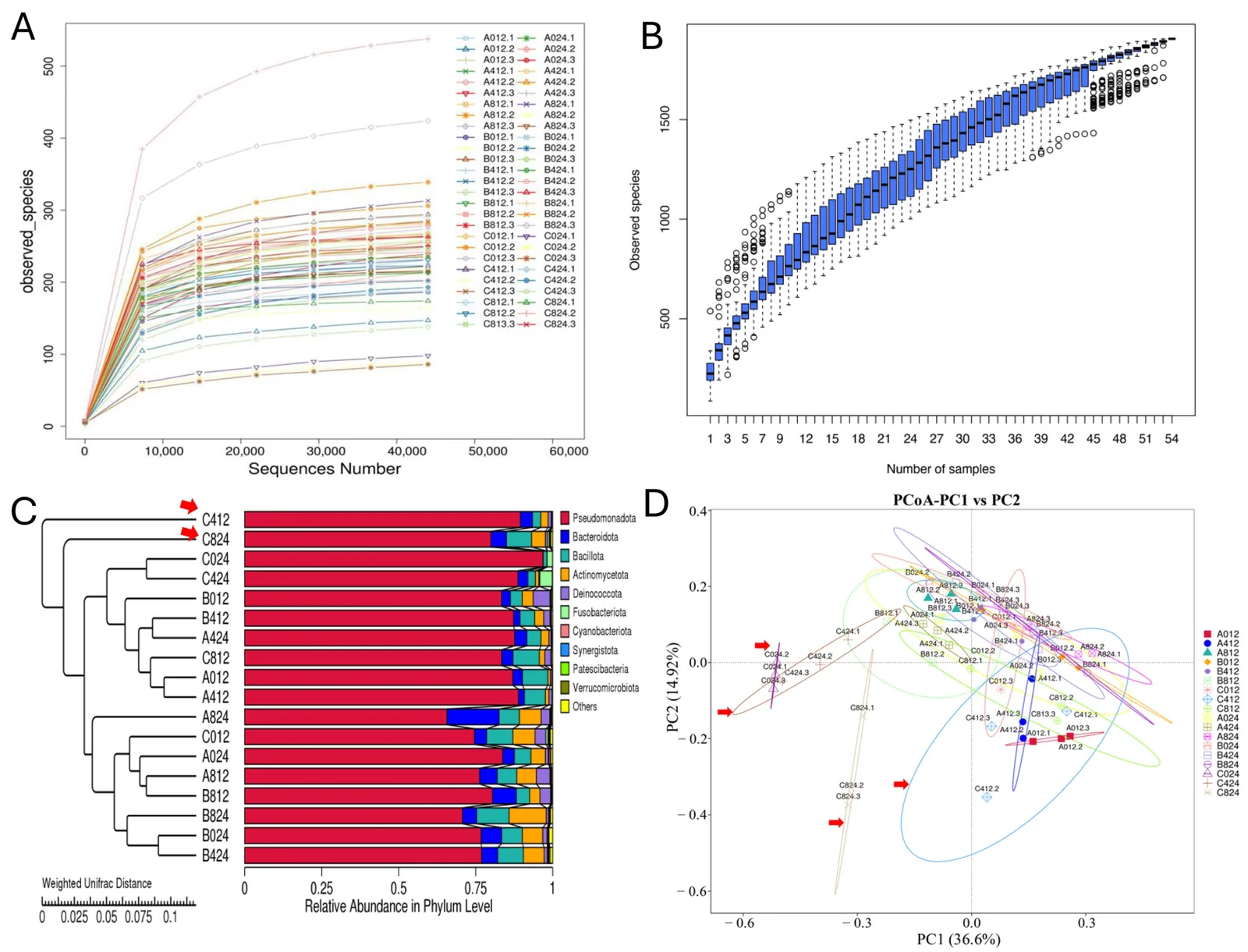
Sequencing quality assessment and microbial community characteristics of bighead carp muscle under coupled temperature and modified atmosphere treatments. (**A**) Rarefaction curves based on observed species for individual samples; (**B**) species accumulation boxplot. The horizontal axis shows the number of samples, and the vertical axis represents the number of observed species; (**C**) UPGMA dendrogram based on weighted UniFrac distance combined with stacked bar chart of phylum-level taxonomic composition; (**D**) PCoA plot based on weighted UniFrac distance. Red arrows indicate samples with high dispersion. Sample naming convention: the prefix letters A, B and C represent MAP formulations containing 40% CO_2_, 60% CO_2_, and 80% CO_2_, respectively. The middle digit (0, 4 and 8) denotes transport-storage temperature (°C), and the final digit (12, 24) indicates transport-storage duration (h). This naming scheme applies to all subsequent figures and tables.

**Figure 2:**
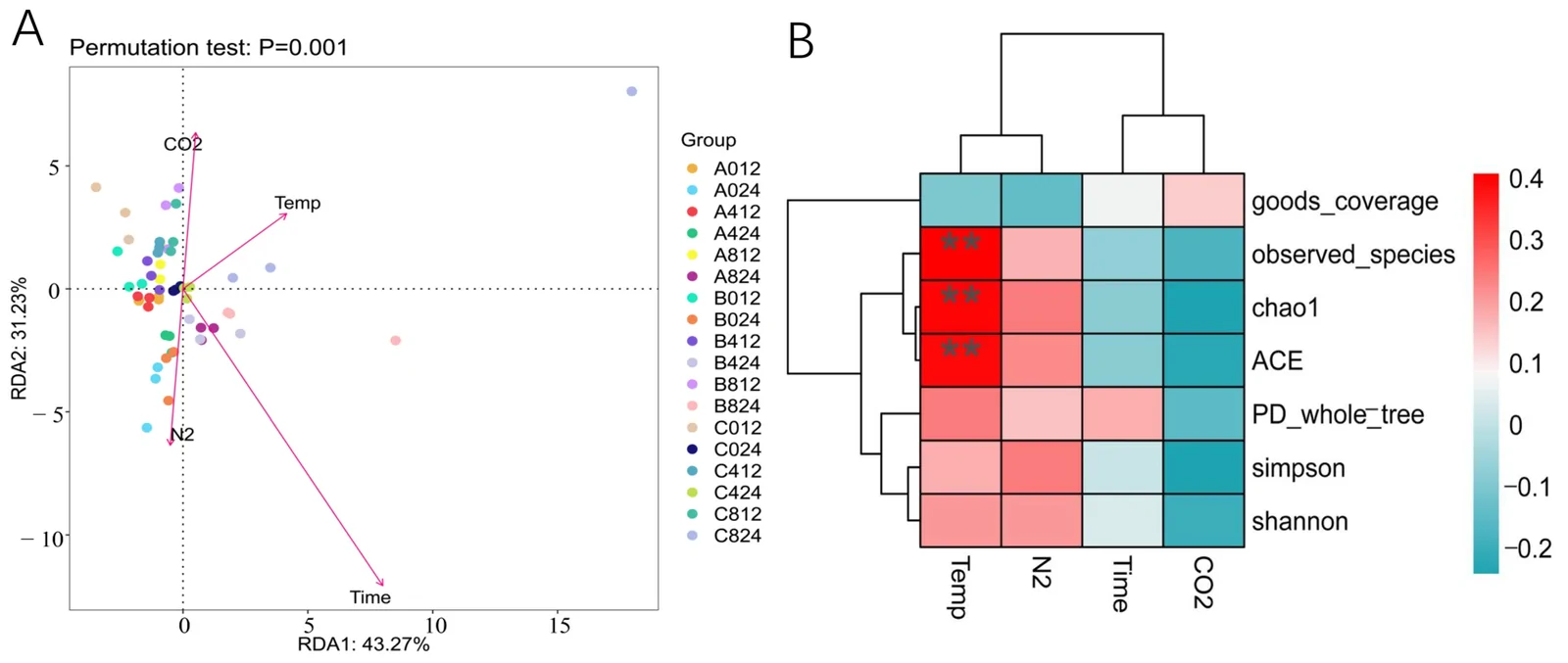
Redundancy analysis (RDA) and Spearman-correlation heatmap between environmental factors and alpha diversity of the microbial communities in bighead carp muscle. (**A**) RDA ordination plot. Colored dots indicate treatment-grouped samples, pink vectors represent environmental factors (Temp: temperature; CO_2_: carbon dioxide concentration; N_2_: nitrogen concentration; Time: transport-storage duration). (**B**) Heatmap of Spearman correlations coefficients between environmental variables and alpha-diversity indices. ** denotes significant correlation with *P* < 0.01.

**Figure 3:**
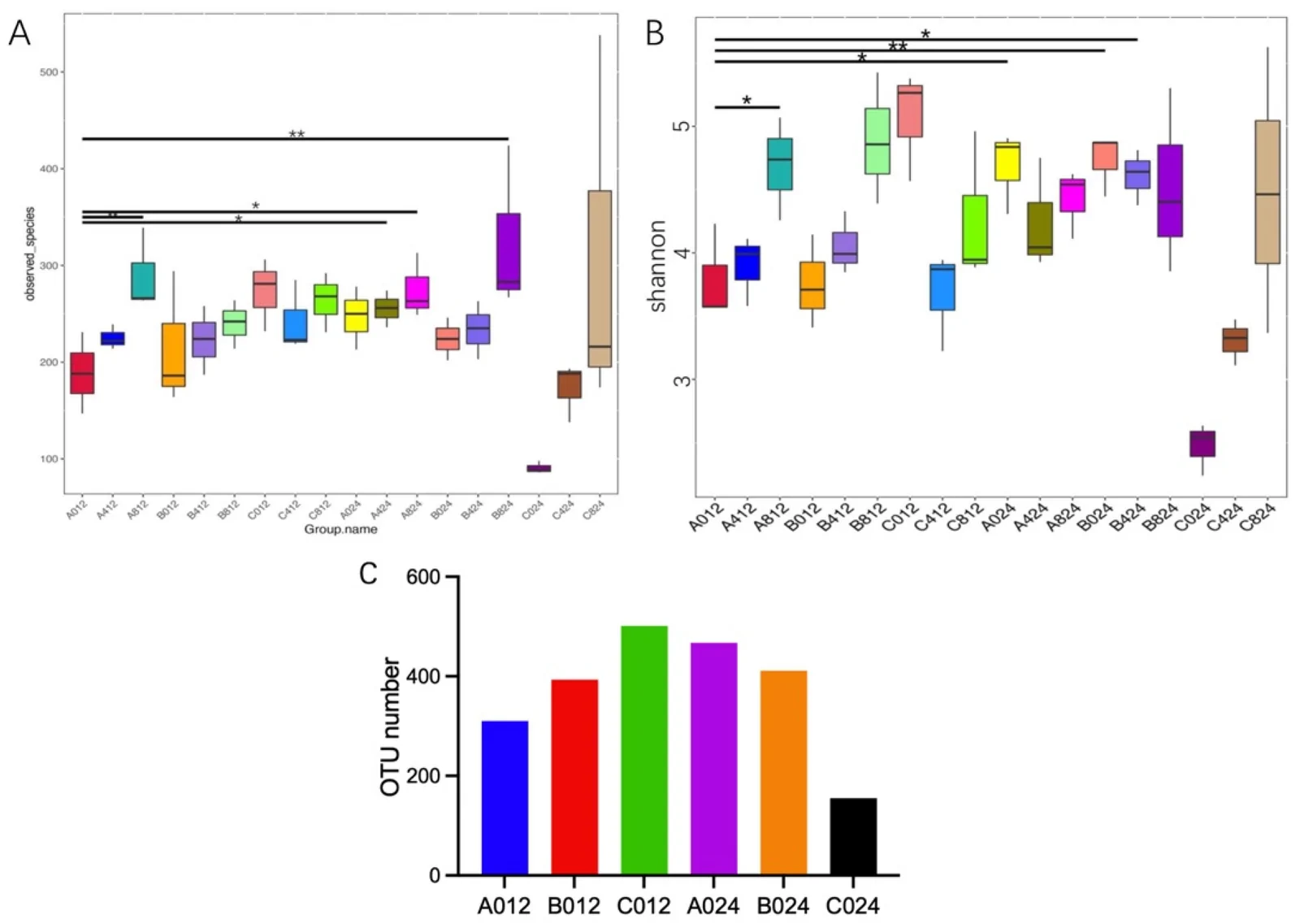
Alpha diversity of microbial communities in bighead carp muscle under combined temperature and MAP treatments. (**A**) Box-plot of observed species index; (**B**) box plot of Shannon index; * and ** denote significant differences at *P* < 0.05 and *P* < 0.01, respectively (Kruskal–Wallis test with pairwise comparisons). (**C**) bar chart of OTU counts for different CO_2_ treatments stored at 0 °C after 12 h and 24 h of simulated transport-storage.

**Figure 4:**
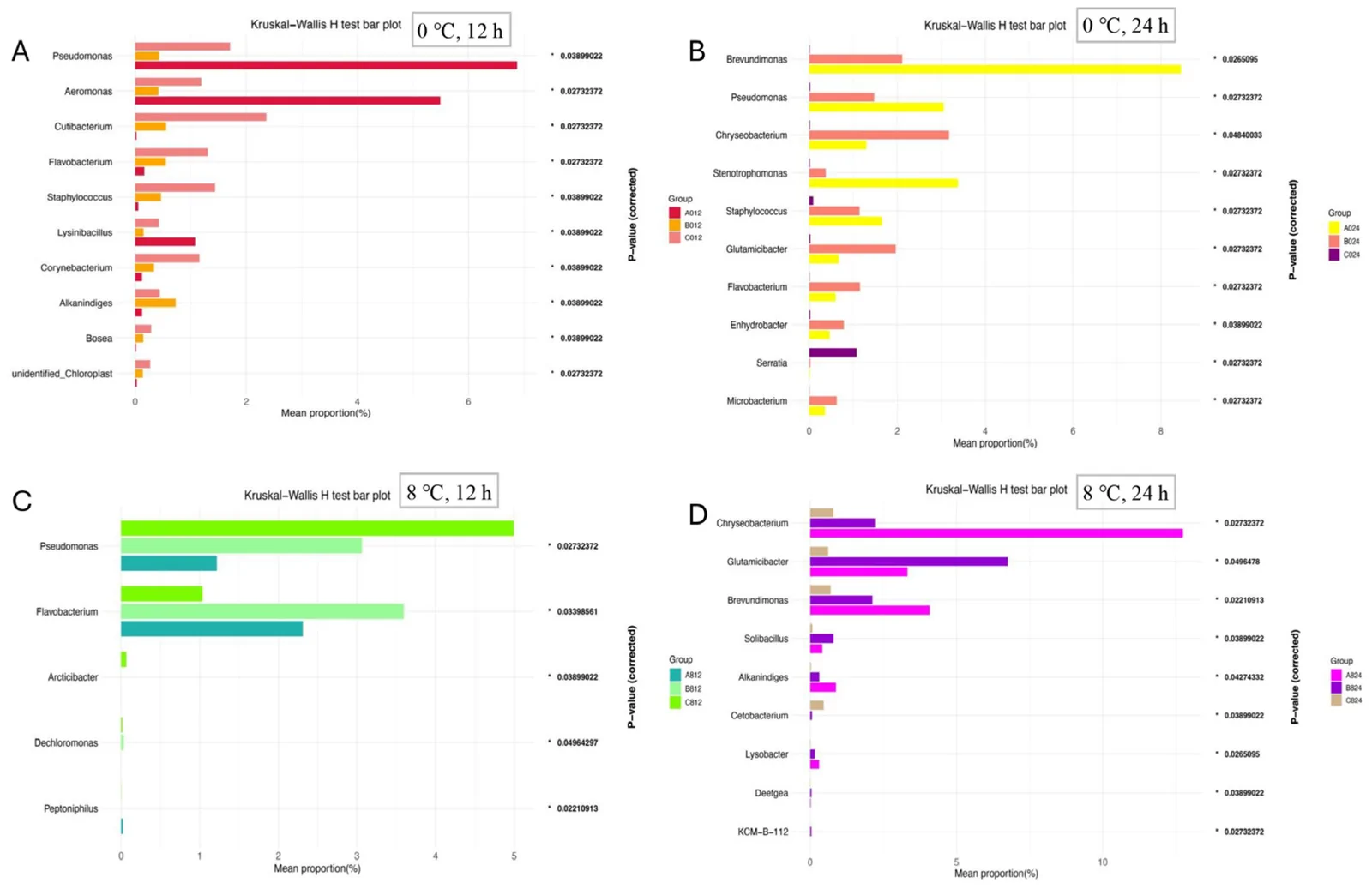
Relative abundance of bacterial genera with significant differences in bighead carp muscle under combined temperature and modified atmosphere treatments, determined using Kruskal–Wallis H test. (**A**) 0 °C 12 h; (**B**) 0 °C 24 h; (**C**) 8 °C 12 h; (**D**) 8 °C 24 h. The x-axis shows the mean relative abundance of bacterial genera, and adjusted *p* values are presented on the right side of each panel.

**Figure 5:**
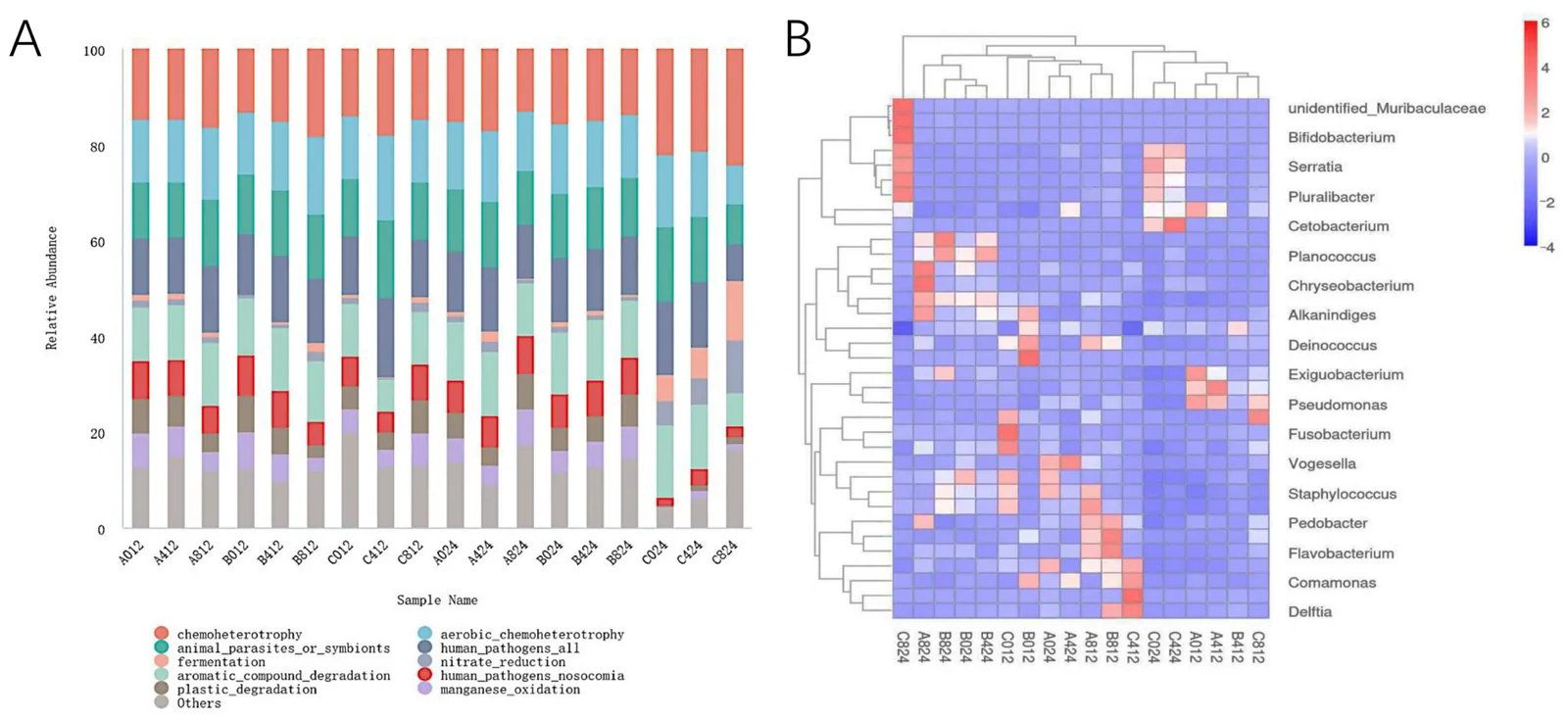
Predicted bacterial functional profiles inferred using PICRUSt2. (**A**) Stacked bar plot illustrating the relative abundance of predicted functional categories across all samples; (**B**) heatmap showing the distribution of predicted functional profiles, with row and column clustering.

## Data Availability

The original contributions presented in this study are included in the article. Further inquiries can be directed to the corresponding authors.

## References

[1] Elsler L.G., Gephart J.A., Zamborain-Mason J., Cashion T., Troell M., Naylor R.L., Bejarano R.A., Golden C.D. (2026). Global nutritional equity of fishmeal and aquaculture trade flows. Proc. Natl. Acad. Sci. USA.

[2] Liu X., Geng R.Y., Do T.N., Lv X., Du X.F. (2024). Transforming China’s Fisheries: A 40-year journey toward sustainable management. Fishes.

[3] Wang Z.C., Wang Q.D., Wang J.L., Wang H., Qian J., Zhang Y.Z., Feng K., Chen Q.Y., Yuan J., Liu J.S. (2022). Evaluation of the control effect of bighead carp and silver carp on cyanobacterial blooms based on the analysis of differences in algal digestion processes. J. Clean. Prod..

[4] Tokarczyk G., Felisiak K., Bienkiewicz G., Adamska I., Przybylska S., Hrebień-Filisińska A., Biernacka P., Boussioud H. (2026). The valorization potential of freshwater fish, including common carp (*Cyprinus carpio*), in the context of obtaining functional food ingredients. Foods.

[5] Zhu W.B., Fu J.J., Luo M.K., Wang L.M., Wang P., Liu Q.G., Dong Z.J. (2022). Genetic diversity and population structure of bighead carp (*Hypophthalmichthys nobilis*) from the middle and lower reaches of the Yangtze River revealed using microsatellite markers. Aquacult. Rep..

[6] Lindstad A.J., Li K.D., Sharmin N., Pettersen M.K. (2025). Suitability of cellulose-based trays with PE/EVOH coating for modified atmosphere packaging of salmon, chicken, and beef. J. Food Sci..

[7] Zhao K., Fu Z., Ji A., Xu D., Xie J., Ding Z. (2026). Modified Atmosphere Packaging (MAP): Flavor regulation of fried shrimp during ice-temperature storage. J. Food Sci..

[8] Moreira-Leite B., Antunes R., Cotas J., Martins N., Costa N., Noronha J.P., Mata P., Diniz M. (2023). Modified Atmosphere Packaging (MAP) for seaweed conservation: Impact on physicochemical characteristics and microbiological activity. Foods.

[9] Li P.Y., Mei J., Xie J. (2023). The regulation of carbon dioxide on food microorganisms: A review. Food Res. Int..

[10] Infante-Neta A.A., D’Almeida A.P., Lima R.S., Cecília J.A., da Silva Junior I.J., Gonçalves L.B., de Albuquerque T.L. (2026). Waste valorization of passion fruit peel hydrolysate for bacterial cellulose production: Influence of nitrogen source on yield and functional properties for food packaging. Foods.

[11] Singh T., Verma P., Deol P., Kumar A., Si C., Dhiman P., Lai C.W. (2026). Trends and advances in development of photodynamic, photothermal, and intelligent food packaging: A review. Compr. Rev. Food Sci. Food Saf..

[12] Miranda G., Berna A., Mulet A. (2019). Dried-fruit storage: An analysis of package headspace atmosphere changes. Foods.

[13] Couvert O., Koullen L., Lochardet A., Huchet V., Thevenot J., Le Marc Y. (2023). Effects of carbon dioxide and oxygen on the growth rate of various food spoilage bacteria. Food Microbiol..

[14] Liu X., Zhang Y., Li D., Luo Y. (2017). Characterization of the microbiota in lightly salted bighead carp (*Aristichthys nobilis*) fillets stored at 4 °C. Food Microbiol..

[15] Liu X., Li D., Li K., Luo Y. (2018). Monitoring bacterial communities in ε-Polylysine-treated bighead carp (*Aristichthys nobilis*) fillets using culture-dependent and culture-independent techniques. Food Microbiol..

[16] Zhuang S., Li Y., Jia S., Hong H., Liu Y., Luo Y. (2019). Effects of pomegranate peel extract on quality and microbiota composition of bighead carp (*Aristichthys nobilis*) fillets during chilled storage. Food Microbiol..

[17] Olatunde O.O., Benjakul S., Vongkamjan K.Y. (2019). Comparative study on nitrogen and argon-based modified atmosphere packaging on microbiological, chemical, and sensory attributes as well as on microbial diversity of Asian sea bass. Food Packag. Shelf Life.

[18] Indriani S., Srisakultiew N., Boonchuen P., Kingwascharapong P., Sai-Ut S., Benjakul S., Pongsetkul J. (2025). Investigating the relationship between microbial community dynamics and flavor profiles in Korat chicken breast fillets under varied packaging conditions. Int. J. Food Microbiol..

[19] Caporaso J.G., Lauber C.L., Walters W.A., Berg-Lyons D., Lozupone C.A., Turnbaugh P.J., Fierer N., Knight R. (2011). Global patterns of 16S rRNA diversity at a depth of millions of sequences per sample. Proc. Natl. Acad. Sci. USA.

[20] Zapala M.A., Schork N.J. (2006). Multivariate regression analysis of distance matrices for testing associations between gene expression patterns and related variables. Proc. Natl. Acad. Sci. USA.

[21] Bolyen E., Rideout J.R., Dillon M.R., Bokulich N.A., Abnet C.C., Al-Ghalith G.A., Alexander H., Alm E.J., Arumugam M., Asnicar F. (2019). Reproducible, interactive, scalable and extensible microbiome data science using QIIME 2. Nat. Biotechnol..

[22] Bai L., Liu M., Sun Y. (2023). Overview of food preservation and traceability technology in the smart cold chain system. Foods.

[23] Bartáková K., Bursova S., Necidová L., Haruštiaková D., Zouharová A., Vorlová L., Klimešová M. (2023). The effect of cold chain disruption on the microbiological profile of chilled fish. J. Microbiol. Biotechnol. Food Sci..

[24] Necidová L., Zouharová A., Haruštiaková D., Bursová Š., Bartáková K., Golian J. (2024). The effect of cold chain disruption on the microbiological profile of chilled chicken meat. Poult. Sci..

[25] Cai Z.C., Wang Y.Y., Liao G.H., Wang J.M. (2024). Effects of cold plasma-activated solution on microbial amounts and diversity of Tilapia fillets at refrigerator storage. LWT.

[26] Møretrø T., Moen B., Heir E., Hansen A., Langsrud S. (2016). Contamination of salmon fillets and processing plants with spoilage bacteria. Int. J. Food Microbiol..

[27] Syropoulou F., Anagnostopoulos D.A., Parlapani F.F., Karamani E., Stamatiou A., Tzokas K., Nychas G.E., Boziaris I.S. (2022). Microbiota succession of whole and filleted European sea bass (*Dicentrarchus labrax*) during storage under aerobic and MAP conditions via 16S rRNA gene high-throughput sequencing approach. Microorganisms.

[28] Jia S., Huang Z., Lei Y.T., Zhang L.T., Li Y., Luo Y.K. (2018). Application of Illumina-MiSeq high throughput sequencing and culture-dependent techniques for the identification of microbiota of silver carp (*Hypophthalmichthys molitrix*) treated by tea polyphenols. Food Microbiol..

[29] Ling Y., Zhou M., Qiao Y., Xiong G., Wei L., Wang L., Wu W., Shi L., Ding A., Li X. (2022). Effects of ozone water combined with ultra-high pressure on quality and microorganism of catfish fillets (*Lctalurus punctatus*) during refrigeration. Front. Nutr..

[30] Knapp B.D., Willis L., Gonzalez C., Vashistha H., Jammal-Touma J., Tikhonov M., Ram J., Salman H., Elias J.E., Huang K.C. (2025). Metabolic rearrangement enables adaptation of microbial growth rate to temperature shifts. Nat. Microbiol..

[31] Jiang X.J., Yao Y.Q., Chen K.Q., Xia X.L., He X. (2026). Temperature dynamics regulate microbial succession and flavor formation in medium-high temperature Daqu. J. Food Compos. Anal..

[32] He Z., Deng Y., Xu M., Li J., Liang J., Xiong J., Yu H., Wu B., Wu L., Xue K. (2020). Microbial functional genes commonly respond to elevated carbon dioxide. Environ. Int..

[33] Xu L., Zhang H., Yue H., Wu S., Yang H., Wang Z., Qi G. (2018). Gas stunning with CO_2_ affected meat color, lipid peroxidation, oxidative stress, and gene expression of mitogen-activated protein kinases, glutathione S-transferases, and Cu/Zn-superoxide dismutase in the skeletal muscles of broilers. J. Anim. Sci. Biotechnol..

[34] Hu W., Shen Y., Ma L., Dai H., Guo T., Zhou H., Yu Y., Zhang Y. (2019). Effects and mechanism of different pretreatment techniques on the quality of refrigerated silver carp meat. Shipin Kexue/Food Sci..

[35] Tsironi T., Ntzimani A., Gogou E., Tsevdou M., Semenoglou I., Dermesonlouoglou E., Taoukis P. (2019). Modeling the effect of active modified atmosphere packaging on the microbial stability and shelf life of gutted sea bass. Appl. Sci..

[36] Feng J., Hu M., Fang C., Benjakul S., Lin H., Zhang B. (2026). Hydrogen-incorporated reducing atmosphere packaging: Effects on protein degradation and microbial diversity of Litopenaeus vannamei. Food Res. Int..

[37] Zhu Z.W., Gao H., Gao T.T., Sun D.W. (2018). Quality comparison of grass carp and salmon fillets packaged in modified atmosphere with different composite films. J. Food Process Eng..

[38] Mursalim M.F., Budiyansah H., Raharjo H.M., Debnath P.P., Sakulworakan R., Chokmangmeepisarn P., Yindee J., Piasomboon P., Elayaraja S., Rodkhum C. (2022). Diversity and antimicrobial susceptibility profiles of *Aeromonas* spp. isolated from diseased freshwater fishes in Thailand. J. Fish. Dis..

[39] Mohamad A., Md Yasin I., Mohd Z., Termizi Y.M., Salleh A., Yunus N.M., Mark L., Shirajum M.M., Zairy Z., Mohammad Noor Azmai A. (2021). The prevalence, putative virulence genes and antibiotic resistance profiles of *Aeromonas* spp. isolated from cultured freshwater fishes in peninsular Malaysia. Aquaculture.

[40] Zhuang S., Tian L., Liu Y., Wang L., Hong H., Luo Y. (2023). Amino acid degradation and related quality changes caused by common spoilage bacteria in chill-stored grass carp (*Ctenopharyngodon idella*). Food Chem..

[41] Zhang C., Zhu F., Jatt A.N., Liu H., Niu L., Zhang L., Liu Y. (2020). Characterization of co-culture of Aeromonas and Pseudomonas bacterial biofilm and spoilage potential on refrigerated grass carp (*Ctenopharyngodon idellus*). Lett. Appl. Microbiol..

